# Impact of urbanization on morbidity of hepatitis A: a national panel study in China during 2005–2018

**DOI:** 10.1186/s40249-023-01104-0

**Published:** 2023-05-25

**Authors:** Bo-Wen Ming, Zhou Yang, Ze-Lin Yan, Chen Shi, Xiao-Han Xu, Li Li, Chun-Quan Ou

**Affiliations:** State Key Laboratory of Organ Failure Research, Department of Biostatistics, Guangdong Provincial Key Laboratory of Tropical Disease Research, School of Public Health, Southern Medical University, Guangzhou, 510515 Guangdong China

**Keywords:** Hepatitis A, Morbidity, Urbanization, Generalized linear mixed model, China

## Abstract

**Background:**

The effect of urbanization on the morbidity of hepatitis A remains unclear. We aimed to estimate the association between various urbanization-related indices and hepatitis A morbidity in China.

**Methods:**

Data on the annual morbidity of hepatitis A, urbanization-related measures (i.e., gross domestic product per capita, the number of hospitalization beds per 1000 persons, illiteracy rate, tap water coverage, motor vehicles per 100 persons, population density, and the proportion of arable land), and meteorological factors in 31 provincial-level administrative divisions of Chinese mainland during 2005–2018 were collected from the National Population and Health Science Data Sharing Platform, China Statistical Yearbooks, and the China Meteorological Data Sharing Service System, respectively. Generalized linear mixed models were applied to quantify the impacts of different urbanization-related indices on the morbidity of hepatitis A in China after adjusting for covariates.

**Results:**

A total of 537,466 hepatitis A cases were reported in China during 2005–2018. The annual morbidity had a decline of 79.4% from 5.64 cases to 1.16 cases per 100,000 people. There were obvious spatial variations with higher morbidity in western China. Nationally, gross domestic product per capita and the number of hospitalization beds per 1000 persons increased from 14,040 to 64,644 CNY and from 2.45 to 6.03 during 2005–2018, respectively. The illiteracy rate decreased from 11.0 to 4.9%. Gross domestic product per capita [relative risk (*RR*) = 0.96, 95% confidence interval (*CI*): 0.92–0.99], and the number of hospitalization beds per 1000 persons (*RR* = 0.79, 95% *CI*: 0.75–0.83) were associated with the declined morbidity of hepatitis A. By contrast, the increased morbidity of hepatitis A was linked to the illiteracy rate (*RR* = 1.04, 95% *CI*: 1.02–1.06). Similar influential factors were detected for children and adults, with greater effects witnessed for children.

**Conclusions:**

People in the western region suffered the heaviest burden of hepatitis A in Chinese mainland. Nationally, there was a sharp decline in the morbidity of hepatitis A. The urbanization process was associated with the reduction of hepatitis A morbidity in China during 2005–2018.

**Graphical Abstract:**

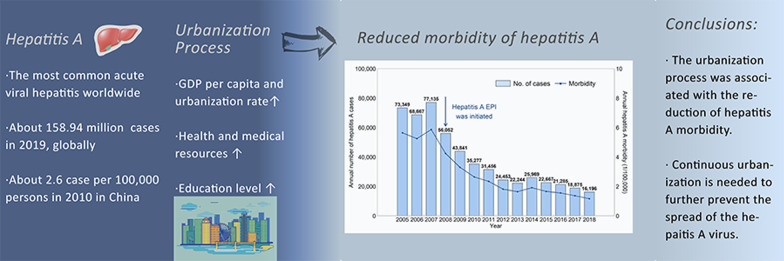

**Supplementary Information:**

The online version contains supplementary material available at 10.1186/s40249-023-01104-0.

## Background

A remarkable urban growth has been witnessed in the past few decades, with people living in towns and cities shooting up from 751 million in 1950 to 4.2 billion in 2018 globally [1]. And the rising trend is expected to maintain. By 2050, approximately seven out of ten individuals will live in urban areas [2]. Urbanization may have two-sided effects on the prevalence of some non-communicable diseases, such as cardiovascular diseases and diabetes, because of changes in dietary intake and physical activities [3]. There are particularly many uncertainties and complexities in the association between increased urbanization and infectious diseases. The economic and environmental development and the improvement in education, sanitation, and healthcare services brought by urbanization facilitate the prevention and control of some infectious diseases [4]. This is the main reason for the remarkable reduction in the global burden of most infectious diseases (e.g., diarrhoeal diseases) over recent decades [5, 6]. On the contrary, urbanization brings changes in the global climate [7], the population density, and the human mobility, which may exacerbate the spread of some infectious diseases like dengue fever and coronavirus disease 2019 [8–11]. The impact of urbanization presents regional heterogeneity. For instance, in The Republic of Korea, people in urban regions with the high population density were likely to be infected with hepatitis A [12]. However, in the United States, hepatitis tended to be prevalent in rural areas [13].

Hepatitis A is the most common acute viral hepatitis worldwide caused by hepatitis A virus (HAV) [14] with estimated 158.94 million cases in 2019 [15]. Understanding the association between urbanization and hepatitis A would have an important implication for the development of prophylactic measures against hepatitis A. However, seldom did previous studies quantify the impact of urbanization on hepatitis A. Three previous studies estimated correlation coefficients between the seroprevalence of hepatitis A and socioeconomic factors such as gross domestic product (GDP) per capita, the proportion of the population with access to clean drinking water, and the value of the human development index [16–18], which did not take the adjustment for other urbanization-related factors into account. Four prior studies estimated the impact of urbanization-related factors, such as the types of water supply, toilets, and residential areas on the seroprevalence of hepatitis A in specific age groups [19–22]. However, these studies focused on children and young adults, and it might not be direct to generalize the conclusion to the whole population because the seroprevalence in elderly people was strikingly different from that in young people [23]. A random effects model was performed to assess the contribution of urbanization to the decline in the mortality of hepatitis A in Chinese mainland during a 5-year period from 1995 to 2010 [24]. Mortality represents the most severe cases. The fatality rate of HAV infection was approximately 0.5% [25]. A study in Korea estimated the effects of district-level urbanization-related indicators on the morbidity of hepatitis A during 2004–2008 [12]. However, the urbanization-related data were available only for a specific year and were not temporally matched with the annual data on the morbidity of hepatitis A. The results mainly explained the contribution of urbanization to the spatial differences instead of temporal variations in the morbidity of hepatitis A. It is of great public health significance to fully examine the effect of urbanization in the sense of the hepatitis A morbidity over a longer period.

China, as the largest developing country, has undergone an unprecedented growth in urbanization [26, 27]. Interestingly, the morbidity of hepatitis A declined during the recent three decades. In 1990, HAV led to approximately 52.6 cases per 100,000 persons [28] and 308.5 thousand disability-adjusted life years (DALYs) in China [29]. With the vigorous urbanization process, the morbidity and DALYs associated with HAV infection dropped to about 2.6 cases per 100,000 persons and 179.1 thousand in 2010 [29, 30]. This change may be in part due to the comprehensive improvement of sanitary facilities related to water supply, excrement disposal, and environmental hygiene [4, 31, 32]. However, the extent to which urbanization contributed to the decline in hepatitis A burden in China warrants further investigations, especially over the last decade when the urbanization process has sped up. Herein, we investigated the effects of several urbanization-related indicators on the morbidity of hepatitis A, in an attempt to provide support for innovative health policymaking.

## Methods

### Data sources

In all regions of Chinese mainland, the reporting and registration of acute hepatitis A cases in the China Information System for Disease Control and Prevention is compulsory [33, 34]. When a hepatitis A-associated public health event occurs, all cases must be notified timely. And the health authority needs to regularly inform the public of the morbidity of all notifiable infectious diseases including hepatitis A. The annual numbers of hepatitis A cases, including clinically-diagnosed and laboratory-confirmed cases [35], reported from 2005 to 2018 across 31 provincial-level administrative divisions (PLADs) in China, were extracted from the National Population and Health Science Data Sharing Platform of the Chinese Center for Disease Control and Prevention [36]. A patient who felt ill to see a physician with relevant clinical symptoms, significantly elevated serum alanine aminotransferase, serum total bilirubin more than double the upper normal limit and/or urine bilirubin positive can be clinically diagnosed with hepatitis A. Laboratory-confirmed cases were defined by any clinically diagnosed patient with tests positive for the IgM antibody to HAV or tests for the IgG antibody to HAV four times of increasing (Additional file 1: Table S1) [30, 37–39]. Age-specific data for children aged < 15 years and adults ≥ 15 years of age were also collected.

Here, we considered multiple urbanization-related indices, including socioeconomic indices (i.e., GDP per capita, the urbanization rate, and the illiteracy rate among adults), infrastructure indices (i.e., the tap water coverage, the number of hospitalization beds per 1000 persons, and motor vehicles per 100 persons), the population density, and the proportion of arable land. These province-level annual data during the study period from 2005 to 2018 were extracted from China Statistical Yearbooks [40]. Meteorological data, including the annual mean temperature, accumulated precipitation, and sunshine duration were downloaded from the China Meteorological Data Sharing Service System [41].

### Statistical analysis

We present the spatial distributions of the hepatitis A morbidity and major urbanization-related indices in 31 PLADs of China. The Global Moran’s *I* test was utilized to determine whether there was a spatial autocorrelation in the morbidity of hepatitis A [42, 43].

We assessed the influential factors of hepatitis A morbidity using univariable analyses and then a multivariable analysis. Specifically, we firstly fitted a generalized linear mixed model (GLMM) for each candidate variable. Next, the Spearman correlation analysis was conducted to explore the pairwise correlation between the variables which were statistically significant in the univariable analyses. If two or more variables were highly correlated (*r*_s_ > 0.8 [44]), the variable with the stronger effect in the univariable analyses was kept for further checking multicollinearity. Then, we calculated the variance inflammation factor (VIF) of the remaining variables, and the ones with the VIF lower than ten were considered in the multivariable analysis [44].

A multivariable GLMM was constructed to assess the effects of the selected variables on the morbidity of hepatitis A, specified as follows:

$$log\left(E\left({Y}_{it}\right)\right)= \, {\alpha }_{i}+{\gamma }_{t}+off set\left(log\left({Pop}_{it}\right)\right)+{\beta }_{1}{GDP}_{it}+{\beta }_{2}{Illiteracy}_{it}+{\beta }_{3}{Water}_{it}+{\beta }_{4}{Bed}_{it}+{\beta }_{5}{Density}_{it}+{\beta }_{6}{Precipitation}_{it}+{\beta }_{7}{Intervention}_{it}$$

where ${Y}_{it}$ denotes the number of hepatitis A cases of the province *i* in the year *t*; *Pop* means the population size; *GDP* means GDP per capita; *Illiteracy* represents the illiteracy rate; *Water* is the tap water coverage; *Bed* means the number of hospitalization beds; *Density* is the population density; *Precipitation* is the annual accumulated precipitation; *Intervention*, standing for the implementation of the Expanded Program on Immunization (EPI), is a categorical variable with 0 and 1 indicating the pre-intervention period and the post-intervention period, respectively. A full cycle of hepatitis A vaccination has been provided for children since the EPI was implemented in each PLAD. Live attenuated vaccines are for children aged ≥ 18 months with one dose, while inactivated vaccines are given in two doses to children ≥ 18 months and ≥ 24 months, respectively [38]. The starting time of the EPI program was not exactly the same date or month for all PLADs but was consistently between late 2008 and 2009. Therefore, in this study based on annual data, we set the starting point as the year of 2009 [45]. Considering the differences in the baseline levels of the hepatitis A morbidity across PLADs and calendar years, we included random-effect intercepts for the province (${\alpha }_{i}$) and the calendar year (${\gamma }_{t}$) in the model. Relative risks (*RR*s) of the hepatitis A morbidity associated with one unit increase in the independent variables and corresponding 95% confidence intervals (*CI*s) were estimated. Additionally, we carried out subgroup analyses to probe into the potential effects of urbanization-related indices in different age groups (i.e., people aged < 15 years and the ones ≥ 15 years).

Sensitivity analyses were conducted to check the robustness of the results. First, we examined the robustness of the results after excluding Tibet from the analysis, because there was a possibility of the under-reporting of hepatitis A in Tibet [46]. Second, we replaced the GDP per capita with the urbanization rate in the multivariable model. These two factors were highly correlated and therefore only one of them was included in the model to avoid the multicollinearity problem. Third, to examine the appropriateness of fitting linear effect in the main analysis, we explored the exposure–response relationships between hepatitis A morbidity and six continuous independent variables, by applying a natural cubic spline (a form of piecewise cubic polynomials) to each of these independent variables separately. Two-sided *P* < 0.05 was considered statistically significant. We used the R statistical software (version 4.2.1, Lucent Technologies, Jasmine Mountain, USA) to perform all analyses.

## Results

### Spatial and temporal distribution of hepatitis A morbidity

**Fig. 1:**
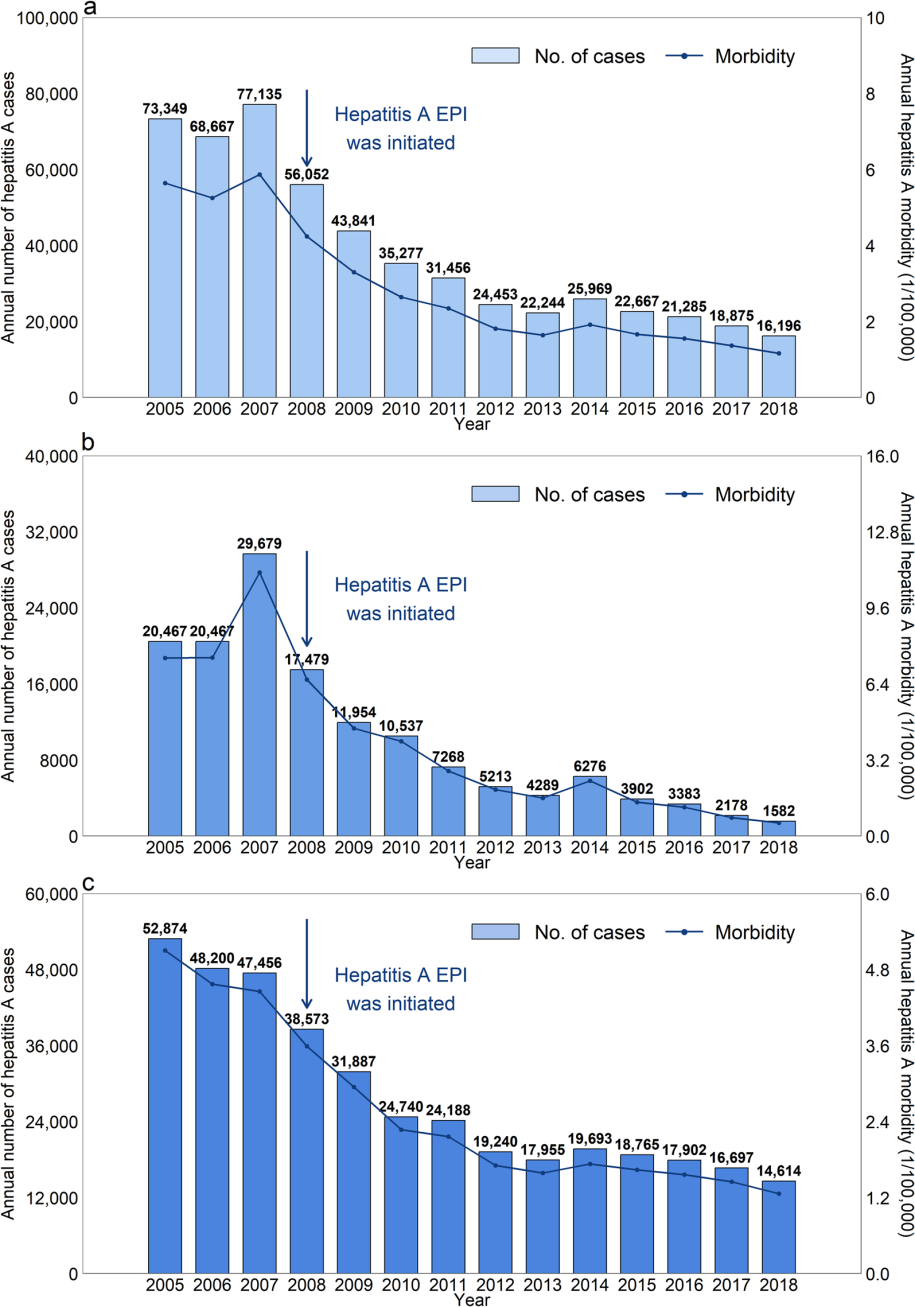
The annual number of cases and morbidity of hepatitis A in 31 provincial-level administrative divisions of China during 2005–2018. **a** The whole population; **b** Children; **c** Adults. *EPI* Expanded Program on Immunization

A total of 537,466 hepatitis A cases including 194 hepatitis A deaths were reported in 31 PLADs of China from 2005 to 2018, with average annual morbidity of 2.89 cases per 100,000 persons (Fig. 1, Additional file 1: Table S2). During the study period, the morbidity of hepatitis A for the whole population declined by 79.4%, from 5.64 cases per 100,000 people in 2005 to 1.16 cases per 100,000 people in 2018. The highest annual morbidity (5.87 cases per 100,000 individuals) was observed in 2007 (Additional file 1: Table S2). Obvious regional differences were observed in the morbidity of hepatitis A, with the higher morbidity in western China (Table 1, Additional file 1: Fig. S1). There was a high spatial correlation across the PLADs, with a Global Moran’s *I* index of 0.25 for the average annual morbidity (Additional file 1: Table S3).

**Table 1:** The average annual hepatitis A morbidity in 31 provincial-level administrative divisions of China

Regions	2005–2018*	2005–2007	2012–2018	Regions	2005–2018	2005–2007	2012–2018
Chinese mainland	2.89	5.59	1.59	Henan	2.12	5.97	0.70
Beijing	0.70	2.02	0.59	Hubei	2.00	4.57	1.55
Tianjin	0.41	0.74	0.34	Hunan	1.24	3.69	0.90
Hebei	0.83	2.29	0.66	Guangdong	1.50	1.99	1.43
Shanxi	2.78	3.44	2.86	Guangxi	2.07	4.09	1.47
Inner Mongolia	1.19	4.22	0.93	Hainan	1.92	8.53	0.99
Liaoning	3.62	4.98	3.46	Chongqing	3.63	9.99	2.87
Jilin	0.89	3.44	0.71	Sichuan	4.34	9.37	3.09
Heilongjiang	0.75	2.43	0.64	Guizhou	2.35	13.58	1.06
Shanghai	1.01	2.29	0.86	Yunnan	3.42	14.60	2.43
Jiangsu	1.03	3.19	0.78	Tibet	6.88	11.92	5.17
Zhejiang	1.24	4.52	0.90	Shaanxi	1.44	4.14	0.97
Anhui	1.60	3.23	1.08	Gansu	8.69	19.00	3.40
Fujian	1.86	3.93	1.36	Qinghai	11.02	15.79	7.57
Jiangxi	1.24	7.11	0.71	Ningxia	3.61	21.76	2.17
Shandong	0.48	1.20	0.46	Xinjiang	15.60	31.77	13.59

^*^The unit of the hepatitis A morbidity is cases per 100,000 persons

### Urbanization-related indices

Nationally, the average annual GDP per capita, illiteracy rate, tap water coverage, number of hospitalization beds per 1000 persons, and population density were 40,200 CNY (USD 5715), 7.4%, 95.0%, 4.25 per 1000 persons, and 447.11 persons per km^2^, respectively (Table 2). Nationally, the GDP per capita and the number of hospitalization beds per 1000 persons increased from 14,040 to 64,644 CNY and from 2.45 to 6.03 during 2005–2018, respectively. The illiteracy rate decreased from 11.0 to 4.9%. The hepatitis A morbidity was positively correlated with the illiteracy rate at the province level (*r*_s_ = 0.57, *P* < 0.001). The GDP per capita, the tap water coverage, the number of hospitalization beds, and the population density generally presented a pattern of low to high from west to east in China, negatively correlated with the morbidity (*r*_s_ = -0.71–-0.47, *P* < 0.001) (Additional file 1: Table S4). However, Tibet was a special case with the moderate morbidity despite a low urbanization level (a low level of GDP per capita, the tap water coverage, the number of hospital beds and the high illiteracy rate). Qinghai had the highest morbidity in adults (Additional file 1: Figs. S2, S3).

**Table 2:** Descriptive statistics of urbanization-related indices and meteorological factors in Chinese mainland during 2005–2018

Variables	Mean	*SD*	Minimum	P_25_	Median	P_75_	Maximum	IQR
GDP per capita (CNY 10,000)	4.02	2.54	0.54	2.09	3.53	5.11	14.02	3.02
Urbanization rate (%)	52.63	14.54	22.61	43.23	50.93	59.28	89.60	16.05
Illiteracy rate (%)	7.43	6.86	1.23	3.68	5.37	8.64	45.65	4.96
Tap water coverage (%)*	95.04	6.59	48.63	92.89	97.29	99.29	123.36	6.40
Hospitalization beds (per 1000 persons)	4.25	1.33	1.65	3.11	4.22	5.16	7.55	2.05
Motor vehicles (per 100 persons)	15.65	10.90	1.54	6.59	13.21	22.17	50.90	15.58
Proportion of arable land (%)	0.03	0.03	0.00	0.02	0.03	0.04	0.46	0.02
Population density (per km^2^)	447.11	644.54	2.24	120.18	286.06	543.46	3818.76	423.29
Average temperature (℃)	13.22	5.63	1.90	9.12	13.55	17.44	25.24	8.32
Accumulated sunshine duration (h)	2079.18	496.76	961.27	1688.27	2043.53	2506.67	2963.22	818.40
Accumulated precipitation (dm)	9.43	5.36	1.13	5.00	8.28	12.91	25.01	7.92

*SD* standard deviation, *P*_*25*_ the 25th percentile, *P*_*75*_ the 75th percentile, *IQR* interquartile range, *GDP* gross domestic product, *CNY* Chinese Yuan

^*^Tap water coverage is defined as the ratio of the number of people who use water to the urban population size multiplied by 100%

### Effects of urbanization-related indices on hepatitis A morbidity

Results of the univariable analyses suggested that the GDP per capita, the urbanization rate, the tap water coverage, the number of hospitalization beds per 1000 persons, the EPI, the population density, and the accumulated precipitation were associated with the decreased risk of hepatitis A infection, while the illiteracy rate was positively associated with the morbidity of hepatitis A (Table 2, Additional file 1: Table S5).

Bivariate Spearman correlation analyses showed that there were high correlations between the GDP per capita, the urbanization rate, and motor vehicles (*r*_s_ > 0.8), implying the multicollinearity among these factors (Additional file 1: Table S4). The final model included the GDP per capita, the illiteracy rate, the tap water coverage, the number of hospitalization beds per 1000 persons, the population density, and the accumulated precipitation, and their values of VIF were all lower than two (Additional file 1: Table S6).

The multivariable analysis revealed a decrease of 6% (*RR* = 0.94, 95% *CI*: 0.91–0.98) in the hepatitis A morbidity associated with an increase of 10,000 CNY in the GDP per capita. The beneficial effects of the number of hospitalization beds per 1000 persons (*RR* = 0.79, 95% *CI*: 0.75–0.83), and the accumulated precipitation (*RR* = 0.98, 95% *CI*: 0.96–1.00) were also observed. The EPI contributed to a 31% reduction in hepatitis A morbidity (*RR* = 0.69, 95% *CI*: 0.61–0.79), while per 1% increase in the illiteracy rate was associated with an increase of 4% in the morbidity of hepatitis A (*RR* = 1.04, 95% *CI*: 1.02–1.06). We did not observe statistically significant effects of population density or tap water coverage after adjusting for other factors in the multivariable analysis (Table 3).

**Table 3:** The effects of various variables on the morbidity of hepatitis A

Variables	Univariable model		Multivariable model	
	*RR*	95% *CI*	*RR*	95% *CI*
GDP per capita (CNY 10,000)	0.74	(0.72–0.76)	0.96	(0.92–0.99)
Illiteracy rate (%)	1.17	(1.15–1.19)	1.04	(1.02–1.06)
Tap water coverage (%)*	0.94	(0.93–0.95)	1.00	(0.99–1.01)
Number of hospitalization beds (per 1000 persons)	0.64	(0.62–0.66)	0.79	(0.75–0.83)
Population density (per km^2^)	1.00	(1.00–1.00)	1.00	(1.00–1.00)
Accumulated precipitation (dm)	0.92	(0.89–0.95)	0.98	(0.96–1.00)
Expanded Program on Immunization	0.34	(0.31–0.37)	0.69	(0.61–0.79)

*GDP* gross domestic product, *RR* relative risk, *95% CI* 95% confidence interval, *CNY* Chinese Yuan

^*^Tap water coverage is defined as the ratio of the number of people who use water to the urban population size multiplied by 100%

### Subgroup analysis

Among all cases, 26.9% were children. The average morbidity of hepatitis A was 3.85 and 2.45 cases per 100,000 persons in children and adults, respectively. The morbidity demonstrated consistent spatial and temporal patterns in children and adults, with the higher morbidity in western China and a persistent decline over time, while a greater reduction was observed among the children than in the adults (92.5% vs 75.0%) (Additional file 1: Table S7 and Fig. S3). In concordant with the findings for all populations, no matter in children or adults, GDP per capita, the number of hospitalization beds per 1000 persons, and the EPI were negatively associated with the morbidity of hepatitis A, while the morbidity increased with the illiteracy rate. The effects were stronger for the child population (Table 4).

**Table 4:** The effects of various variables on the morbidity of hepatitis A in children and adults

Variables	Children		Adults	
	*RR*	95% *CI*	*RR*	95% *CI*
GDP per capita (CNY 10,000)	0.80	(0.74–0.86)	0.96	(0.93–0.99)
Illiteracy rate (%)	1.05	(1.02–1.09)	1.03	(1.02–1.05)
Tap water coverage (%)*	1.01	(1.00–1.03)	0.99	(0.99–1.00)
Number of hospitalization beds (per 1000 persons)	0.69	(0.62–0.76)	0.83	(0.80–0.87)
Population density (per km^2^)	1.00	(1.00–1.00)	1.00	(1.00–1.00)
Accumulated precipitation (dm)	0.95	(0.92–0.99)	0.98	(0.96–0.99)
Expanded Program on Immunization	0.60	(0.46–0.77)	0.73	(0.66–0.81)

*GDP* gross domestic product, *RR* relative risk, *95% CI* 95% confidence interval, *CNY* Chinese Yuan

^*^Tap water coverage is defined as the ratio of the number of people who use water to the urban population size multiplied by 100%

### Sensitivity analyses

The effects of all factors on hepatitis A almost remained the same when Tibet was excluded from the analysis (Additional file 1: Table S8). After replacing the GDP per capita with the urbanization rate in the model, we found that the morbidity of hepatitis A decreased with the urbanization rate (*RR* = 0.97, 95% *CI*: 0.96–0.99) and the estimated effect of other factors changed very little (Additional file 1: Table S9). In accordance with the main results, the exposure–response curves revealed that the logarithm transformation of *RR* changed linearly with GDP per capita, the number of hospitalization beds per 1000 persons, and precipitation, while hepatitis A morbidity monotonically increased with the increasing illiteracy rate. Meanwhile, the associations of the tap water coverage and the population density with the hepatitis A morbidity were statistically non-significant (Additional file 1: Fig. S4).

## Discussion

The present study illustrated the spatial variation in the morbidity of hepatitis A and revealed a negative association between urbanization and the morbidity in China during 2005–2018. We found that the infection of hepatitis A occurred predominately in the west of China, which was consistent with the findings of previous studies [28, 35, 43, 47]. The reason might be that the overall development of western China lags behind the rest of China. The current socioeconomic disparities and the uneven distribution of resources in the west and the rest of China could mirror entrenched public health inequalities [4, 32, 33], which might exert a negative influence on alleviating the national burden of hepatitis A. Therefore, expediting the development of the western region could help further prevent hepatitis A.

Our results demonstrated that the growth of GDP per capita and the reduction of the illiteracy rate were associated with the decreased hepatitis A infection. The findings were consistent with prior studies [4, 16, 17, 22, 24]. People living in areas with the high GDP per capita tend to possess more fortune and resources to maintain better health conditions than those in poverty-stricken areas. Similarly, residents with high education levels may have a better knowledge of the prevention of hepatitis A. The more well-educated people are, the more willing they are to be receptive to health education against hepatitis A and the promotion of personal hygiene practices [4, 48]. In parallel, the progressive urbanization rate is also a prominent urbanization-related index associated with the decreased morbidity. The higher urbanization rate is, the relatively better living and hygienic habits against water-borne communicable diseases people would have [4, 49]. We also noted that the increased access to medical facilities was closely associated with the decreased infection of hepatitis A. The effect of increased hospitalization beds could be attributable to the improvements in sanitation and public-health infrastructures. Under better hygienic conditions, people were not subject to the ingestion of viruses-contaminated food or water, leading to less person-to-person dissemination. The diminution of poverty, the augmentation of public health awareness, and the enrichment of medical resources are essential for the alleviation of the hepatitis A morbidity.

Although hepatitis A morbidity was associated with GDP per capita, illiteracy rates, and the number of hospital beds per 1000 persons in the present study, these urban-related indices cannot fully explain the spatial distribution of hepatitis A morbidity. Especially, compared with the neighboring PLADs (e.g., Xinjiang), Tibet had much lower morbidity despite a lower urbanization level. We cannot rule out the possibility that there were more cases under-reported in Tibet than in other regions [46]. Nevertheless, the sensitivity analysis excluding Tibet indicated that our main results were robust to the data of Tibet. The highest morbidity in adults in Qinghai might be due to the lack of self-protection awareness, especially among peasants and herders with poor hygiene and a low education level. An investigation in 2014 reported that the vaccination coverage of children aged 2–14 was significantly lower in western China than that of other regions (69.1% vs 84.0%) [50], and a national serological survey found a lower anti-HAV antibody seroprevalence in western and central China than in the eastern especially before EPI [51]. This could partly contribute to the high morbidity of hepatitis A in western China. Further research is warranted to understand the differential impacts of EPI on the hepatitis A burden across PLADs. And more efforts should be spared to educate the residents living in locations of high hepatitis A morbidity with information on how to protect themselves from hepatitis A infection, such as practicing environmental, food and personal hygiene.

In addition, we observed that the population density was negatively associated with the morbidity of hepatitis A in the univariable model. However, the population density was not significantly associated with the morbidity of hepatitis A in the multivariable model with the adjustment of GDP per capita. An early study in 1965 in the United States reported that the patterns of the hepatitis morbidity varied with the population density, and the number of infections was much greater in rural than in urban areas with the higher population density [13]. However, a study in the Republic of Korea in 2012 reported the higher morbidity of hepatitis A in areas with the higher population density [12]. Regions with the high population density in different countries are not necessarily economically developed or rich in social resources. In China and the United States, the geographical clustering of areas with the high population density is in the economically developed part of the country where medical and social resources are abundant, whereas, in the Republic of Korea, those with the high density are in the regions with relatively deficient medical resources, which could partially explain the observed differences [12, 13]. Our multivariable analyses indicated that GDP per capita is a better independent socioeconomic indicator influencing hepatitis A than the population density in China.

Finally, age differences were observed in the morbidity of hepatitis A. Children had the higher morbidity of hepatitis A than that of adults and seemed to be more susceptible to the effect of urbanization. During 2005–2008, the morbidity of hepatitis A of children was about 1.5 times that of adults. The reason might be that for lack of health awareness, children were more likely to be exposed to contaminated food, untreated-well water, or springs with inadequate surface water-treatment processes, especially in economically underdeveloped regions. In addition, the average anti-HAV positivity seroprevalence of children was generally lower than that of adults before EPI (51.3% vs 87.4% in 2006 reported by a national serological survey) [51], thus, leaving children susceptible to HAV infection. Additionally, the higher seroprevalence suggested that many adults had once come down with hepatitis A and had acquired lifelong immunity [51]. In 2008, principally because of the marked advancement of urbanization and hepatitis A vaccination for children [47], the morbidity of children significantly decreased with each passing year. However, this beneficial effect of vaccination also acted on adults who did not belong to the target population of vaccination, suggesting indirect protection (e.g., herd immunity). In fact, the morbidity of hepatitis A in adults still decreased slowly in recent years. In 2018, the morbidity in adults was 1.21 per 100,000 persons, which was double that in children (0.56 per 100,000 persons). Thus, preventing hepatitis A in adults is a continuous process requiring the ongoing focus.

This study did suffer from several limitations. First, this study analyzed the fourteen-year data as far as possible, while the time range may not be long enough to detect the long-term role of urbanization in the morbidity of hepatitis A, possibly leading to the underestimation of true effects. Second, the hepatitis A cases were reported with the passive surveillance system, which might miss asymptomatic cases. Thus, our study would underestimate the impact of urbanization-related indices on hepatitis A morbidity. The differences in the incidence of hepatitis A between children and adults may not be adequately elucidated, since HAV infection is more often asymptomatic in young children than in adults and we did not take the prevalence of anti-HAV antibodies into account. Third, this study used the annual data of 31 PLADs in China. Further studies with higher resolution in the spatial dimension, such as the annual data at the city level, would provide more comprehensive information on hepatitis A and urbanization gradients to better evaluate the impact of urbanization on the morbidity of hepatitis A. Finally, we did not reckon with some other unobserved factors which may also affect hepatitis A infection (e.g., dietary habits and health habits of residents in different regions).

## Conclusions

Hepatitis A occurred more frequently in western China compared with other regions of China. The sharp decline of the hepatitis A morbidity was intricately associated with the urbanization process in China, and the effect of urbanization was particularly greater on children. Sustained urbanization is beneficial to further curb the spread of HAV.

## Supplementary Information

**Additional file 1: Table S1.** Diagnostic criteria for viral hepatitis A in Chinese mainland. **Table S2**. Summary statistics for the annual reported hepatitis A in Chinese mainland in 2005–2018. **Table S3.** The Global Moran’s *I* index for the average annual morbidity of hepatitis A in Chinese mainland during 2005–2018. **Table S4.** The Spearman correlation between the morbidity of hepatitis A, urbanization-related indices and meteorological factors. **Table S5.** The univariable analyses of the effects of each urbanization-related index and meteorological factor on the annual morbidity of hepatitis A. **Table S6.** The variance inflammation factor for checking multicollinearity in the main model. **Table S7.** Summary statistics for the annual reported hepatitis A in children and adults in Chinese mainland during 2005–2018. **Table S8.** The effects of urbanization-related indices on the annual morbidity of hepatitis A after excluding the data of Tibet. **Table S9.** The effects of urbanization-related indices including the urbanization rate instead of GDP per capita on the annual morbidity of hepatitis A. **Fig. S1.** The average annual hepatitis A morbidity in the PLADs of Chinese mainland. a The morbidity during 2005–2018; b The morbidity during 2005–2007; c The morbidity during 2012–2018. **Fig. S2.** The average annual urbanization-related indices for each PLAD in Chinese mainland during 2005–2018. a The GDP per capita; b The illiteracy rate; c The tap water coverage; d The number of hospitalization beds; e The population density. **Fig. S3.** The average annual morbidity of hepatitis A in children and adults for the PLADs in Chinese mainland during 2005–2018. a The morbidity in children; b The morbidity in adults. **Fig. S4.** The exposure–response relationship between the morbidity of hepatitis A and each urbanization-related index. Solid lines indicate the point estimates of relative riskof hepatitis A morbidity across values of the six continuous independent variables as compared with 0. The light-blue areas represent the corresponding 95% confidence intervals.

## Data Availability

The datasets generated during and/or analyzed in this study are available from the corresponding authors on reasonable request.

## Abbreviations

HAV: Hepatitis A virus
DALYs: Disability-adjusted life years
GDP: Gross domestic product
PLADs: Provincial-level administrative divisions
GLMM: Generalized linear mixed model
VIF: Variance inflammation factor
EPI: Expanded Program on Immunization
*RR*: Relative risk
95% *CI*: 95% Confidence interval
