# Supplementary material for: Impact of urbanization on morbidity of hepatitis A: a national panel study in China during 2005–2018
Source: Infect Dis Poverty. 2023 May 25;12:56. doi: 10.1186/s40249-023-01104-0 (PMC10208907; doi:10.1186/s40249-023-01104-0)
Supplement: Supplementary file 1 — Additional file 1: Table S1. Diagnostic criteria for viral hepatitis A in Chinese mainland. Table S2. Summary statistics for the annual reported hepatitis A in Chinese mainland in 2005–2018. Table S3. The Global Moran’s I index for the average annual morbidity of hepatitis A in Chinese mainland during 2005–2018. Table S4. The Spearman correlation between the morbidity of hepatitis A, urbanization-related indices and meteorological factors. Table S5. The univariable analyses of the effects of each urbanization-related index and meteorological factor on the annual morbidity of hepatitis A. Table S6. The variance inflammation factor for checking multicollinearity in the main model. Table S7. Summary statistics for the annual reported hepatitis A in children and adults in Chinese mainland during 2005–2018. Table S8. The effects of urbanization-related indices on the annual morbidity of hepatitis A after excluding the data of Tibet. Table S9. The effects of urbanization-related indices including the urbanization rate instead of GDP per capita on the annual morbidity of hepatitis A. Fig. S1. The average annual hepatitis A morbidity in the PLADs of Chinese mainland. a The morbidity during 2005–2018; b The morbidity during 2005–2007; c The morbidity during 2012–2018. Fig. S2. The average annual urbanization-related indices for each PLAD in Chinese mainland during 2005–2018. a The GDP per capita; b The illiteracy rate; c The tap water coverage; d The number of hospitalization beds; e The population density. Fig. S3. The average annual morbidity of hepatitis A in children and adults for the PLADs in Chinese mainland during 2005–2018. a The morbidity in children; b The morbidity in adults. Fig. S4. The exposure–response relationship between the morbidity of hepatitis A and each urbanization-related index. Solid lines indicate the point estimates of relative riskof hepatitis A morbidity across values of the six continuous independent variables as compared with 0. The light-blue [file 40249_2023_1104_MOESM1_ESM.docx]

**Additional file**

**Supplementary Tables**

**Table S1.** Diagnostic criteria for viral hepatitis A in Chinese mainland.

**Table S2**. Summary statistics for the annual reported hepatitis A in Chinese mainland in 2005–2018.

**Table S3.** The Global Moran’s *I* index for the average annual morbidity of hepatitis A in Chinese mainland during 2005–2018.

**Table S4.** The Spearman correlation between the morbidity of hepatitis A, urbanization-related indices and meteorological factors.

**Table S5.** The univariable analyses of the effects of each urbanization-related index and meteorological factor on the annual morbidity of hepatitis A.

**Table S6.** The variance inflammation factor for checking multicollinearity in the main model.

**Table S7.** Summary statistics for the annual reported hepatitis A in children and adults in Chinese mainland during 2005–2018.

**Table S8.** The effects of urbanization-related indices on the annual morbidity of hepatitis A after excluding the data of Tibet.

**Table S9.** The effects of urbanization-related indices including the urbanization rate instead of GDP per capita on the annual morbidity of hepatitis A.

**Supplementary Figures**

**Fig. S1.** The average annual hepatitis A morbidity in the PLADs of Chinese mainland. a The morbidity during 2005–2018; b The morbidity during 2005–2007; c The morbidity during 2012–2018.

**Fig. S2.** The average annual urbanization-related indices for each PLAD in Chinese mainland during 2005–2018. a The GDP per capita; b The illiteracy rate; c The tap water coverage; d The number of hospitalization beds; e The population density.

**Fig. S3.** The average annual morbidity of hepatitis A in children and adults for the PLADs in Chinese mainland during 2005–2018. a The morbidity in children; b The morbidity in adults.

**Fig. S4.** The exposure-response relationship between the morbidity of hepatitis A and each urbanization-related index. Solid lines indicate the point estimates of relative risk (*RR*) of hepatitis A morbidity across values of the six continuous independent variables as compared with 0. The light-blue areas represent the corresponding 95% confidence intervals.

**Table S1.** Diagnostic criteria for viral hepatitis A in Chinese mainland.

|  | **Diagnostic criteria of viral hepatitis A** |
| --- | --- |
| Epidemiology | 1.1 The history of having contaminated food or contaminated water within 2–7 weeks before the onset of the disease, or the history of close contact with acute hepatitis A patients, or a local outbreak or epidemic of hepatitis A, or the travel history to hepatitis A endemic areas |
| Clinical symptoms | 2.1 Gastrointestinal symptoms such as fever, fatigue and poor appetite, nausea, vomiting, bloating, constipation, and hepatomegaly with tenderness or tapping pain  2.2 Jaundice of sclera and skin with the exclusion of other illnesses |
| Laboratory tests | 3.1 Significantly elevated serum alanine aminotransferase (ALT)  3.2 Serum total bilirubin (TBIL) more than double the upper normal limit and/or urine bilirubin positive  3.3 Tests positive for the IgM antibody to HAV or tests for the IgG antibody to HAV four times of increasing |
| Diagnose | Clinically diagnosed cases:  1.1+2.1+2.2+3.1 or 1.1+2.1+2.2+3.1+3.2 or 2.1+2.2+3.1 or 2.1+2.2+3.1+3.2  Confirmed cases:  Clinically diagnosed cases+3.3 |

**Table S2**. Summary statistics for the annual reported hepatitis A in Chinese mainland in 2005–2018.

| Year | No. of  cases | Morbidity  (per 100,000) | No. of  deaths | Mortality  (per 10,000,000) | Case fatality  rate (per 100,000) |
| --- | --- | --- | --- | --- | --- |
| 2005 | 73,349 | 5.64 | 43 | 0.33 | 58.62 |
| 2006 | 68,667 | 5.25 | 37 | 0.28 | 53.88 |
| 2007 | 77,135 | 5.87 | 29 | 0.22 | 37.60 |
| 2008 | 56,052 | 4.24 | 10 | 0.08 | 17.84 |
| 2009 | 43,841 | 3.30 | 21 | 0.16 | 47.90 |
| 2010 | 35,277 | 2.64 | 4 | 0.03 | 11.34 |
| 2011 | 31,456 | 2.35 | 13 | 0.10 | 41.33 |
| 2012 | 24,453 | 1.81 | 5 | 0.03 | 20.45 |
| 2013 | 22,244 | 1.64 | 2 | 0.01 | 8.99 |
| 2014 | 25,969 | 1.92 | 8 | 0.06 | 30.81 |
| 2015 | 22,667 | 1.66 | 10 | 0.07 | 44.12 |
| 2016 | 21,285 | 1.55 | 5 | 0.04 | 23.49 |
| 2017 | 18,875 | 1.37 | 4 | 0.03 | 21.19 |
| 2018 | 16,196 | 1.17 | 3 | 0.02 | 18.52 |
| Total | 537,466 | 2.89 | 194 | 0.10 | 31.15 |

**Table S3.** The Global Moran’s *I* index for the average annual morbidity of hepatitis A in Chinese mainland during 2005–2018.

| Year | Moran’s *I* index | *P* |
| --- | --- | --- |
| 2005 | 0.49 | < 0.001 |
| 2006 | 0.51 | < 0.001 |
| 2007 | 0.39 | < 0.001 |
| 2008 | 0.55 | < 0.001 |
| 2009 | 0.50 | < 0.001 |
| 2010 | 0.45 | < 0.001 |
| 2011 | 0.55 | < 0.001 |
| 2012 | 0.67 | < 0.001 |
| 2013 | 0.63 | < 0.001 |
| 2014 | 0.25 | < 0.001 |
| 2015 | 0.41 | < 0.001 |
| 2016 | 0.20 | < 0.001 |
| 2017 | 0.42 | < 0.001 |
| 2018 | 0.53 | < 0.001 |
| Annual average | 0.25 | < 0.001 |

**Table S4.** The Spearman correlation between the morbidity of hepatitis A, urbanization-related indices and meteorological factors.

|  | Morbidity | GDP | Urban | Illiteracy | Water | Bed | Vehicle | Intervention | Agriculture | Density | Temperature | Sunshine | Precipitation |
| --- | --- | --- | --- | --- | --- | --- | --- | --- | --- | --- | --- | --- | --- |
| Morbidity | 1 |  |  |  |  |  |  |  |  |  |  |  |  |
| GDP | -0.71 | 1 |  |  |  |  |  |  |  |  |  |  |  |
| Urban | -0.62 | 0.87 | 1 |  |  |  |  |  |  |  |  |  |  |
| Illiteracy | 0.57 | -0.60 | -0.66 | 1 |  |  |  |  |  |  |  |  |  |
| Water | -0.47 | 0.68 | 0.61 | -0.42 | 1 |  |  |  |  |  |  |  |  |
| Bed | -0.47 | 0.75 | 0.57 | -0.46 | 0.48 | 1 |  |  |  |  |  |  |  |
| Vehicle | -0.62 | 0.88 | 0.67 | -0.48 | 0.60 | 0.83 | 1 |  |  |  |  |  |  |
| Intervention | -0.48 | 0.59 | 0.32 | -0.40 | 0.35 | 0.61 | 0.68 | 1 |  |  |  |  |  |
| Agriculture | -0.46 | 0.17 | 0.22 | -0.25 | 0.17 | -0.01 | -0.04 | 0.02 | 1 |  |  |  |  |
| Density | -0.50 | 0.45 | 0.53 | -0.43 | 0.49 | 0.08 | 0.24 | 0.03 | 0.68 | 1 |  |  |  |
| Temperature | -0.15 | 0.09 | 0.13 | -0.05 | 0.11 | -0.19 | -0.08 | 0.00 | 0.38 | 0.49 | 1 |  |  |
| Sunshine | 0.12 | 0.02 | -0.01 | -0.01 | 0.05 | 0.18 | 0.20 | 0.01 | -0.49 | -0.41 | -0.82 | 1 |  |
| Precipitation | -0.21 | 0.09 | 0.13 | -0.05 | 0.04 | -0.16 | -0.08 | 0.05 | 0.39 | 0.38 | 0.88 | -0.85 | 1 |

Abbreviations: Morbidity, hepatitis A morbidity; GDP, gross domestic product per capita; Illiteracy, illiteracy rate among adults; Water, tap water coverage; Bed, the number of hospitalization beds per 1000 persons; Vehicle, vehicles per 100 persons; Intervention, expansion of immunization program; Agriculture, proportion of arable land; Density, population density; Temperature, average ambient temperature; Precipitation, accumulated precipitation; Sunshine, accumulated sunshine duration.

**Table S5.** The univariable analyses of the effects of each urbanization-related index and meteorological factor on the annual morbidity of hepatitis A.

| Variables | *RR* | 95% *CI* |
| --- | --- | --- |
| GDP per capita (10,000 CNY) | 0.74 | (0.72–0.76) |
| Urbanization rate (%) | 0.91 | (0.90–0.91) |
| Illiteracy rate (%) | 1.17 | (1.15–1.19) |
| Tap water coverage (%) | 0.94 | (0.93–0.95) |
| Number of hospitalization beds (per 1000 persons) | 0.64 | (0.62–0.66) |
| Vehicles (per 100 persons) | 0.95 | (0.94–0.95) |
| Implementation of immunization program | 0.34 | (0.31–0.37) |
| Proportion of arable land (%) | 0.06 | (0.00–1.42) |
| Population density (per km^2^)^a^ | 1.00 | (1.00–1.00) |
| Average ambient temperature (℃) | 0.96 | (0.91–1.01) |
| Accumulated precipitation (dm) | 0.92 | (0.89–0.95) |
| Accumulated sunshine duration (h) | 1.00 | (1.00–1.00) |

Abbreviation: GDP, gross domestic product; *RR*, relative risk; 95% *CI*, 95% confidence interval.

**Table S6.** The variance inflammation factor for checking multicollinearity in the main model.

|  | GDP | Illiteracy | Water | Bed | Density | Precipitation | Intervention |
| --- | --- | --- | --- | --- | --- | --- | --- |
| VIF | 1.25 | 1.13 | 1.01 | 1.28 | 1.35 | 1.02 | 1.06 |

Abbreviations: VIF, variance inflammation factor; GDP, gross domestic product per capita; Illiteracy, illiteracy rate among adults; Water, tap water coverage; Bed, the number of hospitalization bed per 1000 persons; Precipitation, accumulated precipitation; Intervention, expansion of immunization program.

**Table S7.** Summary statistics for the annual reported hepatitis A in children and adults in Chinese mainland during 2005–2018.

|  | Children | |  | Adults | |
| --- | --- | --- | --- | --- | --- |
| Year | No. of  cases | Morbidity  (per 100,000) |  | No. of  cases | Morbidity  (per 100,000) |
| 2005* | 20,467 | 7.49 |  | 52,874 | 4.83 |
| 2006 | 20,467 | 7.51 |  | 48,200 | 4.36 |
| 2007 | 29,679 | 11.09 |  | 47,456 | 4.23 |
| 2008 | 17,479 | 6.59 |  | 38,573 | 3.40 |
| 2009 | 11,954 | 4.54 |  | 31,887 | 2.78 |
| 2010 | 10,537 | 4.00 |  | 24,740 | 2.14 |
| 2011 | 7268 | 2.74 |  | 24,188 | 2.07 |
| 2012 | 5213 | 1.96 |  | 19,240 | 1.64 |
| 2013 | 4289 | 1.61 |  | 17,955 | 1.52 |
| 2014 | 6276 | 2.33 |  | 19,693 | 1.66 |
| 2015 | 3902 | 1.43 |  | 18,765 | 1.58 |
| 2016 | 3383 | 1.22 |  | 17,902 | 1.50 |
| 2017 | 2178 | 0.78 |  | 16,697 | 1.39 |
| 2018 | 1582 | 0.56 |  | 14,614 | 1.21 |
| Total | 144,674 | 3.85 |  | 392,784 | 2.45 |

* In 2005, there were eight cases whose ages were missing.

**Table S8.** The effects of urbanization-related indices on the annual morbidity of hepatitis A after excluding the data of Tibet.

|  | Univariable model | |  | Multivariable model | |
| --- | --- | --- | --- | --- | --- |
|  | *RR* | 95% *CI* |  | *RR* | 95% *CI* |
| GDP per capita (10,000 CNY) | 0.74 | (0.72–0.76) |  | 0.96 | (0.92–1.00) |
| Illiteracy rate (%) | 1.20 | (1.18–1.22) |  | 1.04 | (1.02–1.06) |
| Tap water coverage (%) | 0.93 | (0.92–0.94) |  | 1.00 | (0.99–1.00) |
| Number of hospitalization beds (per 1000 persons) | 0.64 | (0.62–0.66) |  | 0.79 | (0.75–0.83) |
| Population density (persons per km^2^) | 1.00 | (1.00–1.00) |  | 1.00 | (1.00–1.00) |
| Accumulated precipitation (dm) | 0.92 | (0.89–0.95) |  | 0.97 | (0.95–0.99) |
| Expanded Program on Immunization | 0.34 | (0.30–0.37) |  | 0.71 | (0.62–0.82) |

Abbreviations: *RR*, relative risk; 95% *CI*, 95% confidence interval.

**Table S9.** The effects of urbanization-related indices including the urbanization rate instead of GDP per capita on the annual morbidity of hepatitis A.

|  | Univariable model | |  | Multivariable model | |
| --- | --- | --- | --- | --- | --- |
|  | *RR* | 95% *CI* |  | *RR* | 95% *CI* |
| Urbanization rate (%) | 0.91 | (0.90–0.91) |  | 0.97 | (0.96–0.99) |
| Illiteracy rate (%) | 1.17 | (1.15–1.19) |  | 1.03 | (1.01–1.05) |
| Tap water coverage (%) | 0.94 | (0.93–0.95) |  | 1.00 | (0.99–1.01) |
| Number of hospitalization beds (per 1000 persons) | 0.64 | (0.62–0.66) |  | 0.85 | (0.79–0.91) |
| Population density (persons per km^2^) | 1.00 | (1.00–1.00) |  | 1.00 | (1.00–1.00) |
| Accumulated precipitation (dm) | 0.92 | (0.89–0.95) |  | 0.98 | (0.96–1.00) |
| Expanded Program on Immunization | 0.34 | (0.31–0.37) |  | 0.66 | (0.58–0.74) |

Abbreviations: *RR*, relative risk; 95% *CI*, 95% confidence interval.

**
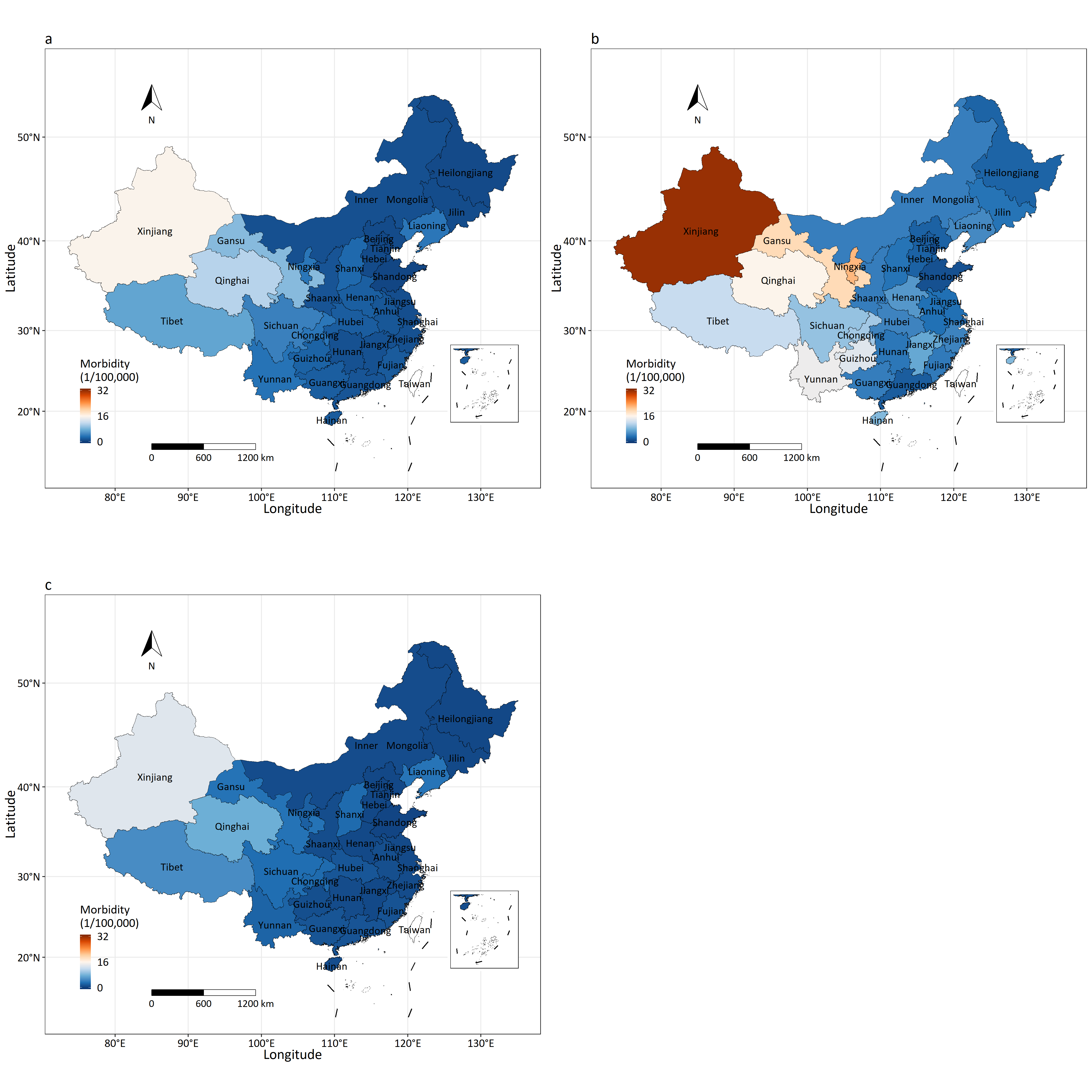
**

**Fig. S1.** The average annual hepatitis A morbidity in the PLADs of Chinese mainland. a The morbidity during 2005–2018; b The morbidity during 2005–2007; c The morbidity during 2012–2018.

**
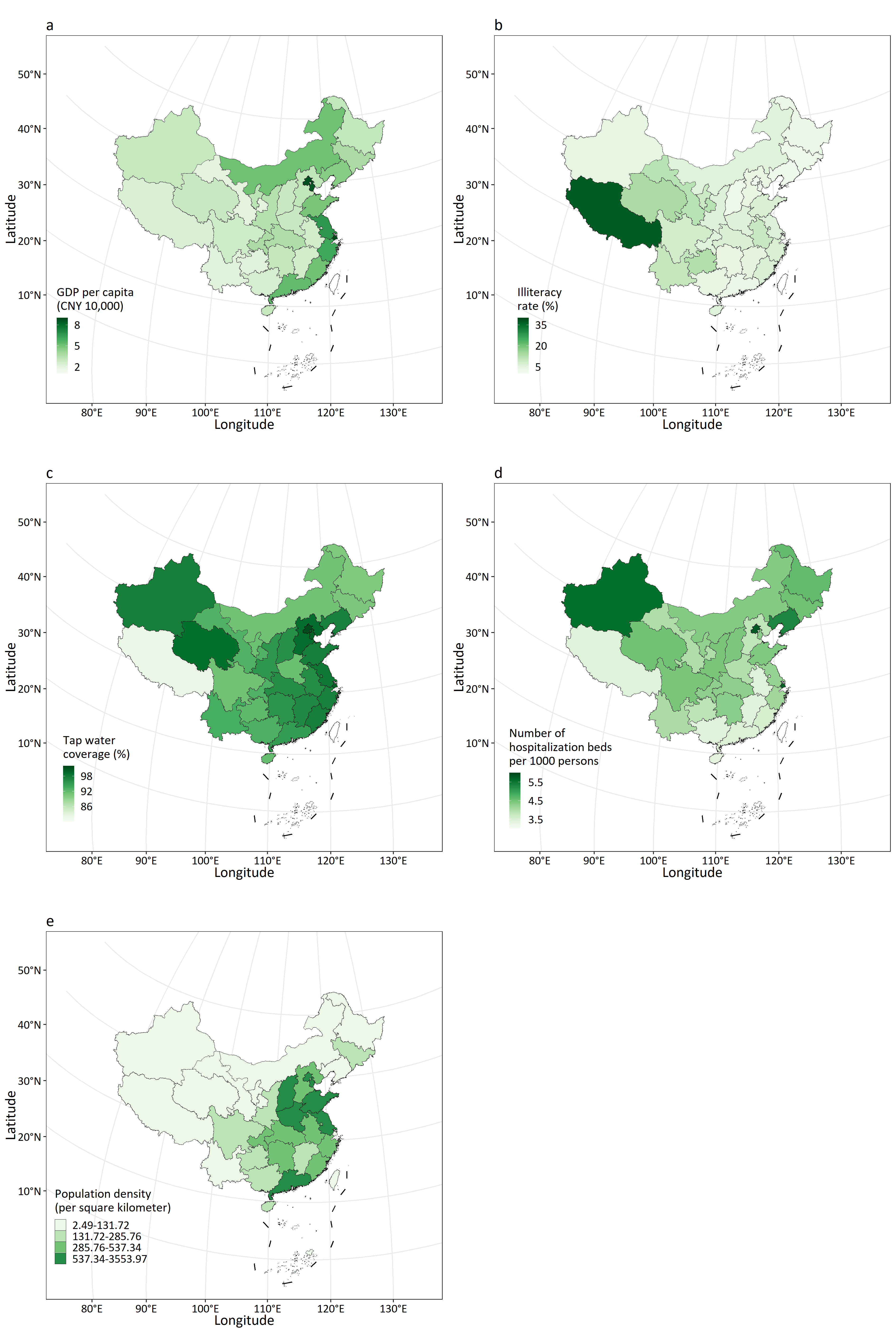
**

**Fig S2.** The average annual urbanization-related indices for each PLAD in Chinese mainland during 2005–2018. a The GDP per capita; b The illiteracy rate; c The tap water coverage; d The number of hospitalization beds; e The population density.


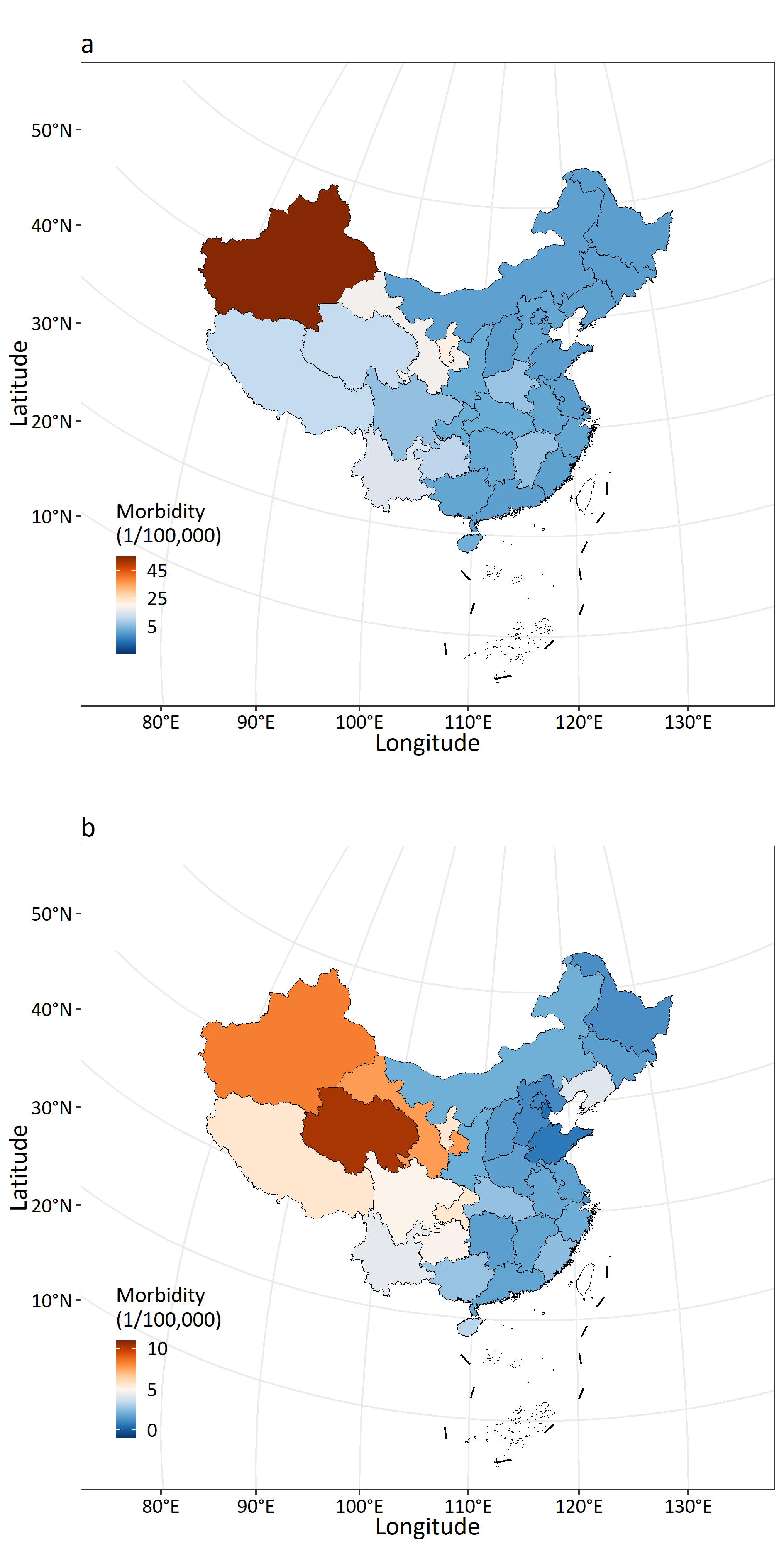


**Fig S3.** The average annual morbidity of hepatitis A in children and adults for the PLADs in Chinese mainland during 2005–2018. a The morbidity in children; b The morbidity in adults.


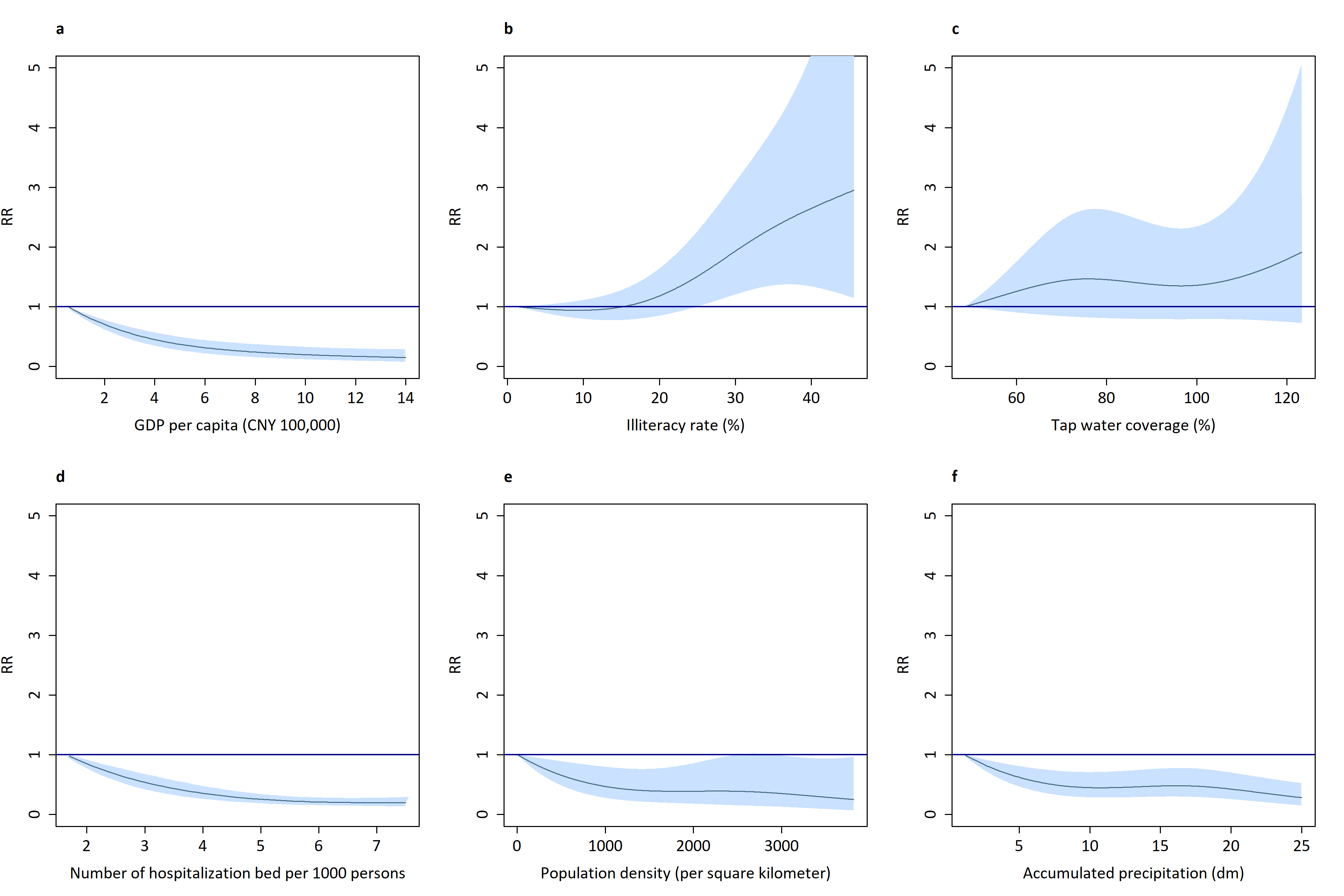


**Fig S4.** The exposure-response relationship between hepatitis A morbidity and each urbanization-related index. Solid lines indicate the point estimates of relative risk (*RR*) of hepatitis A morbidity across values of the six continuous independent variables as compared with 0. The light-blue areas represent the corresponding 95% confidence intervals.
